# Enhancement of X-ray emission from nanocolloidal gold suspensions under double-pulse excitation

**DOI:** 10.3762/bjnano.9.242

**Published:** 2018-10-01

**Authors:** Wei-Hung Hsu, Frances Camille P Masim, Armandas Balčytis, Hsin-Hui Huang, Tetsu Yonezawa, Aleksandr A Kuchmizhak, Saulius Juodkazis, Koji Hatanaka

**Affiliations:** 1 Research Center for Applied Sciences, Academia Sinica, Nankang, Taipei 11529, Taiwan; 2 Centre for Micro-Photonics, Swinburne University of Technology, Hawthorn, VIC 3122, Australia; 3 Division of Materials Science and Engineering, Faculty of Engineering, Hokkaido University, Sapporo, Hokkaido 0608628, Japan; 4 School of Natural Sciences, Far Eastern Federal University (FEFU), Vladivostok 690041, Russia; 5 Institute of Automation and Control Processes, Far Eastern Branch, Russian Academy of Science, Vladivostok 690041, Russia; 6 Melbourne Centre for Nanofabrication, the Victorian Node of the Australian National Fabrication Facility, Clayton 3168 VIC, Australia; 7 College of Engineering, Chang Gung University , Guishan, Taoyuan 33302, Taiwan; 8 Department of Materials Science and Engineering, National Dong-Hwa University, Shoufeng, Hualien 97401, Taiwan

**Keywords:** double pulse, gold nanoparticles, intense femtosecond laser, plasma, water, X-ray

## Abstract

Enhancement of X-ray emission was observed from a micro-jet of a nano-colloidal gold suspension in air under double-pulse excitation of ultrashort (40 fs) near-IR laser pulses. Temporal and spatial overlaps between the pre-pulse and the main pulse were optimized for the highest X-ray emission. The maximum X-ray intensity was obtained at a 1–7 ns delay of the main pulse irradiation after the pre-pulse irradiation with the micro-jet position shifted along the laser beam propagation. It was revealed that the volume around gold nanoparticles where the permittivity is near zero, ε ≈ 0, accounts for the strongest absorption, which leads to the effective enhancements of X-ray emission.

## Introduction

X-ray-related science and technology is one of the most powerful tools for material science, in particular regarding nanomaterials [1]. Femtosecond laser-based X-ray pulse sources [2] have been available on the basis of linear- and nonlinear-optical processes such as plasma formation [3–5] and ablation [6]. In practical applications for X-ray diffraction [7] and X-ray absorption fine structure (XAFS) measurements [8–9], or further nonlinear X-ray processes, a high flux of X-ray pulses is indispensable. X-ray intensity enhancements can be expected through an effective increase of the laser-absorption efficiency and an enhanced generation of high-energy electrons from a highly ionized plasma with high electron temperature [10–11] under intense laser irradiation.

For an efficient augmentation in X-ray intensity, femtosecond (fs)-laser excitation of metal nanoparticles and nanoclusters is of great interest, due to the efficient conversion of the absorbed energy into energetic ions, electrons and X-rays [12–14]. Plasmonic nanoparticles are expected to be highly useful for femtosecond laser-based X-ray emission due to high functionality, large absorption cross section and spectral selectivity based on surface plasmon resonance [15]. An increase of the laser-absorption efficiency can be expected in plasmonic nanoparticles, which results in the efficient generation of highly ionized charge states [13–14]. It is expected that this characteristic interaction between intense femtosecond laser and plasmonic nanoparticles will contribute to a broad field of applications ranging from pulsed X-ray generation to energetic particle sources [14] or terahertz wave emission from aqueous solutions [16].

In recent experimental studies, intense fs-laser excitation of plasmonic gold nanoparticles with different sizes was applied for the efficient X-ray generation in an aqueous phase [17–18], and plasmonic silver nanoparticles were excited to accelerate electrons in vaccum [19]. In addition, there is another new theoretical approach on X-ray sources using plasmonic graphene [20]. Nanoparticles of silver [21] or aluminum [22] could be also considered. However, experimental approaches have been limited to flat samples [23] so far. Gold, with the higher atomic number and a high stability even in aqueous environments, should be considered a main target for fs-laser-induced X-ray experiments especially in aqueous solution.

Recently, it was reported that fs-double-pulsed excitation (including pump–probe type experiments) with dielectrics amplifies optical emission [24]. This double-pulsed excitation is considered to be another way to efficiently augment the X-ray intensity [25]. A significant enhancement in X-ray intensity from a water film was observed under double-pulse excitation with a pre-pulse ahead of the main pulse [26–27]. The pre-pulse irradiation with relatively low intensity induces a time-dependent laser-induced plasma (in the range of picoseconds) and ablation (in the range of nanoseconds). At a certain time delay, Δ*t*, after the pre-pulse irradiation, the main-pulse irradiation of the solution surface results in enhanced X-ray generation. Investigation of X-ray generation intensity and spectrum in distilled water revealed a significant variation of the optimum position of the solution surface for different Δ*t* up to 15 ns [28]. Analyses of the relation between the time-dependent X-ray generation and the optimum laser irradiation position are still missing for other aqueous solutions, especially for experiments under ambient pressure. Previous studies, mostly with solid targets in vacuum, using metallic targets at high intensities of more than 10^15^ W/cm^2^ have shown strong X-ray generation enhancement when a pre-pulse was used. This was explained through the bremsstrahlung of hot electrons and plasma waves breaking at the peak X-ray intensities [29–31].

Here, we explore the optimization of enhancement in X-ray generation from an aqueous solution flow of plasmonic gold nanoparticles under fs-double-pulse excitation in air. The optimization of the solution film surface position with respect to the focal position of irradiation was systematically investigated for the most intense generation of hard X-rays.

## Experimental

Colloidal suspensions of gold nanospheres were prepared via kinetically controlled seeded-growth synthesis of citrate-stabilized nanoparticles [32–34]. In a 250 mL three-necked round-bottomed flask, 2.2 mM sodium citrate in deionized water (*>*18 MΩ, 150 mL) was heated at 115 °C for 15 min under vigorous stirring. After it reached the boiling point, 1 mL of HAuCl_4(aq)_ (25 mM) was added. The color of the solution changed from yellow to bluish gray and to light pink over a period of 10 min. Immediately after the synthesis of the gold seed solution, the temperature was cooled down to 90 °C and the seeded growth of gold nanospheres was carried out. 1 mL of HAuCl_4(aq)_ solution (25 mM) was injected in the reaction vessel. The reaction was finished after 30 min and the process was repeated twice. The sample was diluted by extracting 55 mL of the sample, adding 53 mL of deionized water and 2 mL of 60 mM sodium citrate. By changing the volume in each growth step, it is possible to control the particle concentration. The diameter of gold nanoparticles was 20 nm and the concentration was ca. 1.4 × 10^−7^ mol/cm^3^ or ρ ≈ 3.5 × 10^11^ nanoparticles/cm^3^. These values correspond to an intra-colloidal separation of 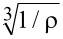 = 1.4 μm or 1.07 × 10^2^ nanoparticles in the focal volume approximated by a cylinder of volume π*w*^2^·2*z*_R_.

**Figure 1:**
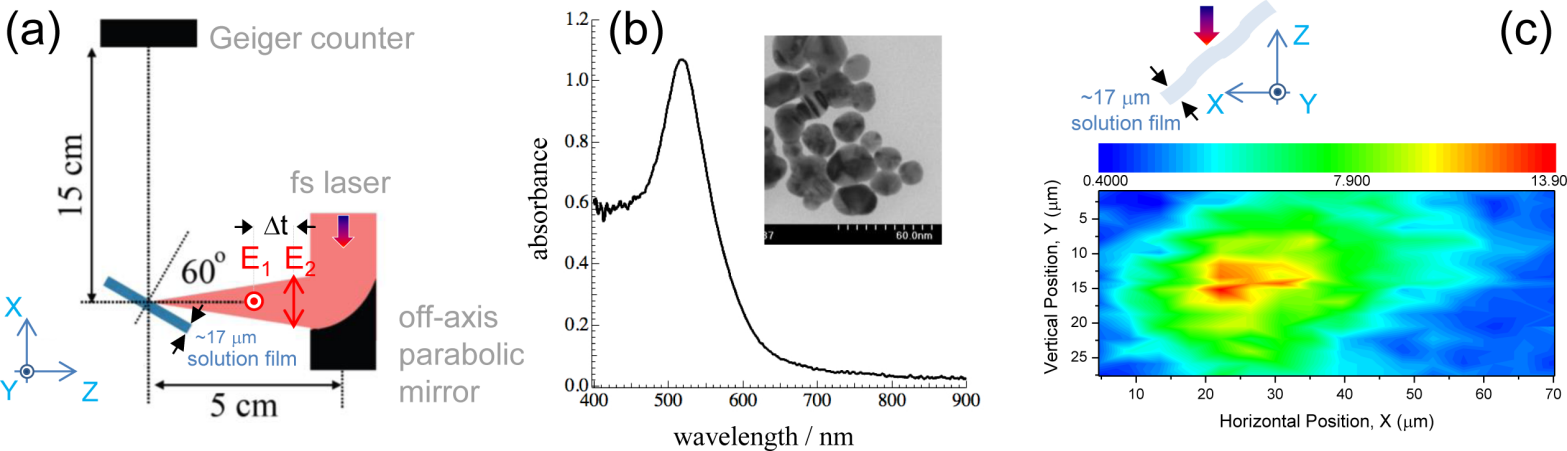
(a) Schematics of experiment showing polarizations of the pre-pulse *E*_1_ and the main pulse *E*_2_ in the plane of incidence (*xz*-plane). (b) The absorption spectrum of the sample gold solution with an SEM image of gold nanoparticles. (c) Beam steering optimization in *xy*-projection for the highest yield of X-ray generation at 100 ps delay between the pre-pulse (energy *E*_1_ = 80 μJ) and the main pulse (*E*_2_ = 700 μJ). The distance between water film and Geiger counter was 15 cm, with an aperture of 2 mm. The orientation of the *E*_1_ polarization is shown for reference.

The micro-jet positioning system has been introduced in our earlier studies [28,35]. A beam-steering system for the pre-pulse irradiation was additionally installed to the setup used in this study. Figure 1 shows the principle and the optimization map in the lateral plane perpendicular to the laser beam. A precision of 1 μm for the axial displacement of the solution film with respect to the incoming laser beam was achieved within less than 1 s, which is satisfactory for practical applications of X-ray generation [35].

Transform-limited fs-laser pulses *t*_p_ = 40 fs with central wavelength λ = 800 nm at 1 kHz repetition rate were horizontally polarized (Mantis, Legend Elite, HE USP, Coherent, Inc.). Two pulses were combined collinearly for the double-pulse irradiation using half-wave plates (65-906, Edmund Optics) and polarization beam splitters (47-048, Edmund Optics). Vertically and horizontally polarized pulses are defined as the pre-pulse and the main pulse, respectively. The vertically polarized pre-pulse intensity is below the threshold of X-ray generation, which is obtained only under double-pulse irradiation. The delay of the main pulse relative to the pre-pulse, Δ*t*, was controlled within three time zones: 0–5 ns (I), 5–10 ns (II) and 10–15 ns (III), which were set into the optical path of the pre-pulse. The optical delay for each time zone is obtained with an 80 cm long variable delay line (SGSP 46-800, Sigma Koki). Change of the time zones from I to II (or III) was made with two independent fixed retroreflectors. The optical path of the main pulse had the same optical length as the longest optical path of the pre-pulse. After combination, the pulses were tightly focused onto a film from the solution jet with an off-axis parabolic mirror (effective focus length of *f* = 50.8 mm, reflection angle of 90°, and numerical aperture NA =0.25, 47-097, Edmund Optics) in air. Under these focusing conditions, the theoretical/geometrical diameter of the focal spot is *2w*_0_ = 1.22λ/NA = 3.9 μm and the axial extent along the propagation estimated as two Rayleigh lengths *2z*_R_ = 2*n*λ/(NA^2^) = 25.5 μm where *n* = 1 is the refractive index of air. The focal region is longer than the spatial extent of the pulse *ct*_p_ = 12 μm, *c* being the speed of light. A 17 ± 2 μm thick jet-flow of a gold nanocolloidal suspension was used as a target. The incident angle of the laser was set at θ = 60° to the normal of the surface of the solution flow. This corresponded to a twice larger effective thickness of the solution film *h*/cosθ ≡ 2*h* encountered by the laser beam. Usually, more than 120 mL of sample solution was being circulated by a pump during experiments. This amount is much larger than the volume of the laser irradiation at the focus, which can be estimated to be 1.5 × 10^−4^ mL. An apparent degradation of the solution absorption spectrum was not observed after experiments. It has been already reported, under similar experimental conditions, that the energy conversion efficiency of the laser pulse to hard X-rays, the electron temperatures, and X-ray photon numbers are estimated to be about 10^−8^ [25], 3–10 keV [27], and 3.8 × 10^10^ photons s^−1^ 4πsr^−1^ [26], respectively.

## Theory: Epsilon-Near-Zero (ENZ)-Material

A strong generation of X-rays is related to a large amount of absorbed energy and a high temperature [3–4]. In terms of relative permittivity (dielectric constant), ε_r_, a transparent medium (water) with colloidal gold nanoparticles represents an effective medium with an overall positive real part of the permittivity. Upon excitation, the material is approaching dielectric breakdown and a state of ε_r_ ≈ 0 (epsilon-near-zero (ENZ) state; 
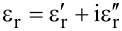
). Under the ENZ condition, the strongest absorption takes place [36–37]:

[1]
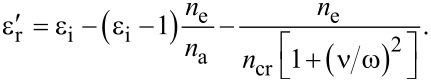


Here, ε_i_ is the intrinsic permittivity of material, ν is the electron–ion collision rate, ω is the optical cyclic frequency at the wavelength of excitation, *n*_e,a,cr_ are the electron, atom and critical densities, respectively. The imaginary part of the permittivity is given by:

[2]
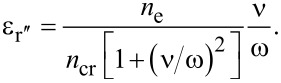


The threshold fluence for water to reach the ENZ state can be calculated from the consideration that all absorbed energy density is converted to thermal energy of electrons. It can be calculated from the ablation threshold expression of a dielectric [37]:

[3]
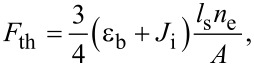


where *l*_s_ = *c*/(κω) is the skin depth related to the imaginary part of the refractive index 
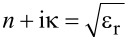
, *c* is the speed of light in vacuum, *A* is the absorbance, ε_b_ is the binding energy [eV/atom], and *J*_i_ is the ionization potential. The threshold fluence for the creation of the ENZ state is calculated for full ionization, i.e., with the bandgap energy Δ_g_ in Equation 3 instead of *(ε*_b_ + *J*_i_). For water, Δ_g_ = 9.5 eV. However, the creation of a solvated electron out of the valence band requires only 6.2 eV, which was used in the estimations [38]. The ENZ state is created when the electron density *n*^ENZ^ ≈ ε_i_*n*_cr_ (Equation 1); for water ε_i_ = n^2^ = 1.77, defined by the refractive index *n* = 1.33 at the excitation wavelength λ = 800 nm. The critical plasma density is *n*_cr_ = ω^2^*m*_e_m*ε_0_/*e*^2^, where *m*_e_ is the electron mass, m* = 1 is its effective mass in the plasma, *e* is the electron charge, ε_0_ is the permittivity of vacuum, and ω = 2π*c*/λ is the cyclic frequency of light. This expression can be used to calculate the corresponding plasma frequency ω_p_ when the electron density, *n*_e_, is known. For instance, for gold *n*_e_ = 5.9 × 10^22^ cm^−3^.

The refractive index under ENZ conditions can be calculated as 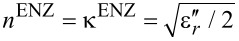 with use of Equation 2:


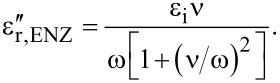


Then *A*^ENZ^ = 4κ/[(κ + 1)^2^ + κ^2^] and the fluence threshold for the ENZ state is (Equation 3):


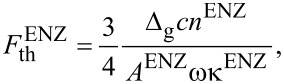


with Δ_g_ = 6.2 eV for a solvated electron and an electron collision rate for momentum exchange ν = 10^15^ s^−1^. The following numerical values are valid for the ENZ state in water: *n*^ENZ^ = κ^ENZ^ = 0.86, *A*^ENZ^ = 0.82, 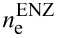 = 3.1 × 10^21^ cm^−3^, 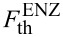 = 42 mJ/cm^2^. This corresponds to the ionization threshold for water through the creation of solvated electrons. When the bandgap energy of water Δ_g_ = 9.5 eV is taken as the ionization threshold, a slightly larger fluence of 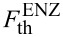 = 60 mJ/cm^2^ will result.

For the metal (gold nanoparticles) we consider the ablation threshold expression equivalent to Equation 3 in which binding energy and electron work function (escape energy) need to be accounted for through (ε_b_ + ε_esc_) together with the substitution *A*/*l*_s_ = 4π/λ. For the ionization threshold, the electron work function is considered. Then, the ionization threshold reads [37]:


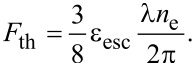


For gold the following values are valid: ε_esc_ = 5.1 eV and 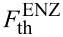 = 230 mJ/cm^2^.

The above presented analysis takes into account the host material and the electron–ion plasma through the definition of permittivity, where the ENZ region defines the strongest light absorption. Due to the fast interaction, the permittivity conditions corresponding to the ENZ are changing in time and space.

## Results and Discussion

### Two-pulse excitation for X-ray emission

**Figure 2:**
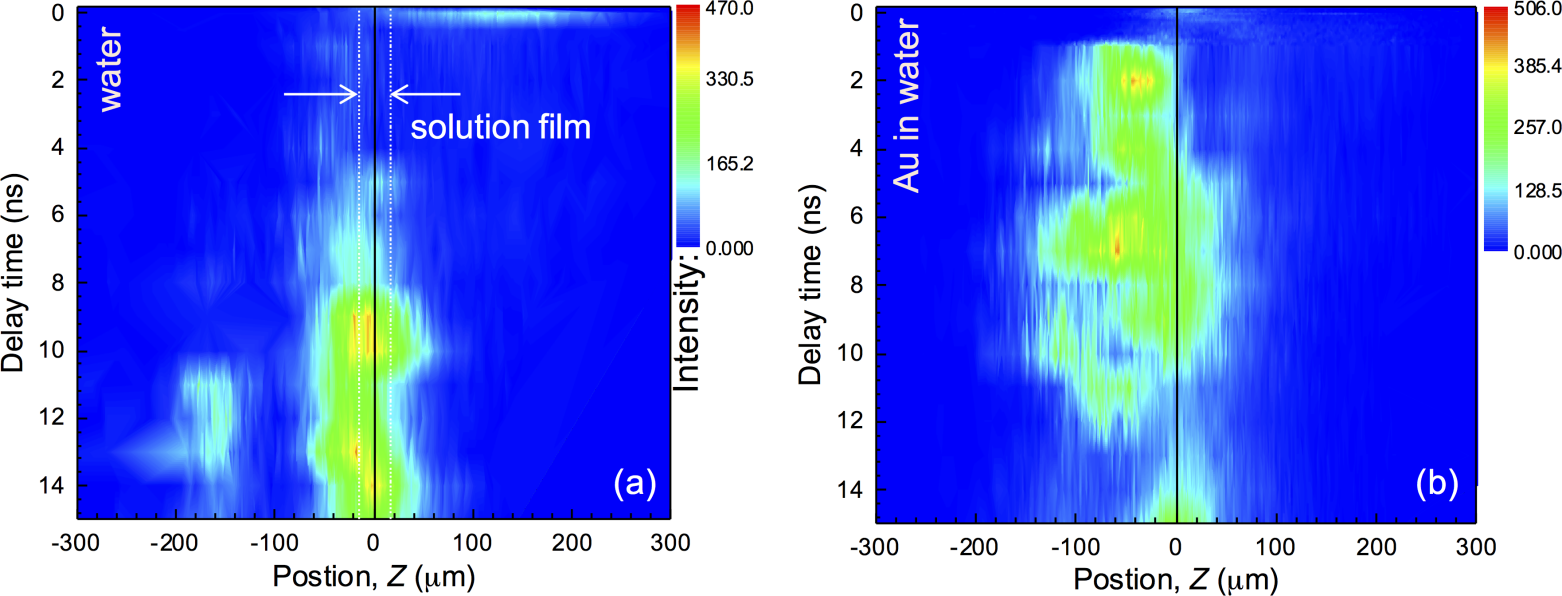
X-ray intensities from (a) a water film and (b) a film of a colloidal suspension of gold nanospheres irradiated by a pre-pulse at 80 μJ/pulse and a main pulse at 700 μJ/pulse with different time delays. The automatic positioning system was used to find the maximum X-ray intensity [35]. The laser irradiates the solution film from the right side in these figures. The width of the solution film hit by the laser pulse at an incidence angle of θ = 60° is *2h* ≈ 40 μm (shown in panel (a)).

**Figure 3:**
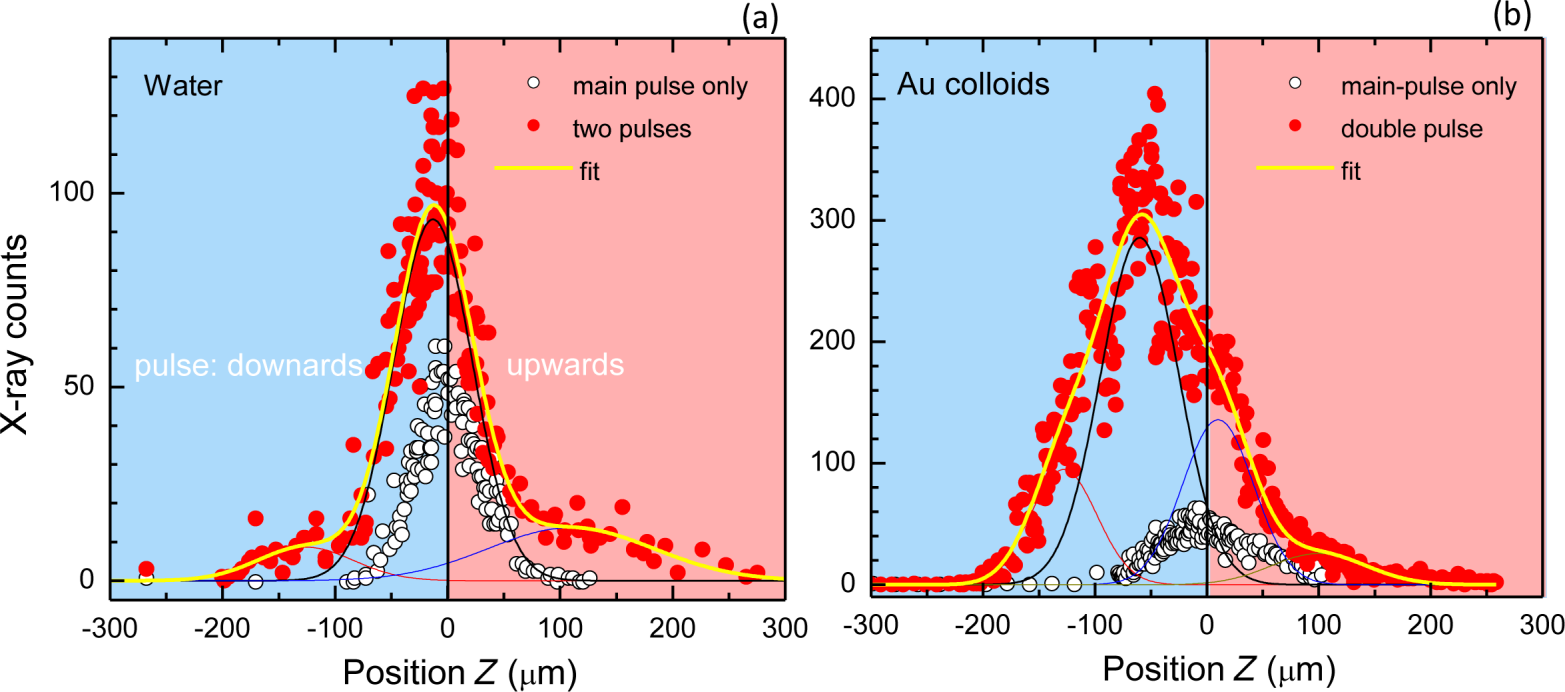
X-ray intensity at different positions of the water film (a) and the gold nanosphere colloidal solution film (b) at Δ*t* = 6 ns. The laser irradiates the solution film from the right side in these figures. The fit constitutes a multi-Gaussian convolution. The intensity of the pre-pulse is 80 μJ. Under double-pulsed excitation, the spatial profiles of X-ray intensity are significantly different in the cases of water and the gold nanospheres colloidal solution.

Figure 2 shows the X-ray generation as a function of the time between the pre-pulse of 80 μJ and the main pulse of 700 μJ. Strong X-ray generation was observed at a delay of 1 ns in the case of the gold colloid. For the distilled water film, the required time was ca. 8 ns. Comparison of X-ray generation from water and colloidal gold solution at a time delay of Δ*t* = 6 ns is shown in Figure 3. For the nanocolloidal gold suspension, a film shifted downwards by ca. 60 μm along the propagation of the laser beam (the negative side of the *z*-axis shown in Figure 3b) caused the strongest X-ray generation, which was approximately 5 to 6 times larger that during single-pulse exposure. This shift reflects the time integrated X-ray signal from the region of the strongest absorption due to ENZ conditions multiplied with the mass density of the solution. The shift of the solution film position was only ca. 10 μm (half of the film thickness) for distilled water. The strongest X-ray generation from pure water was about a third for the double-pulse irradiation. A long delay of 1–10 ns for intense X-rays from the colloidal solution film is consistent with the formation and growth of bubbles around gold nanoparticles and their ablation as discussed in the following.

The absorption of laser energy by the gold nanoparticles is expected to be relatively small considering their volume fraction and off-resonance excitation conditions. The plasmon resonance excitation for these NPs is expected to be near 520 nm. This provides a moderate enhancement of the optical near-fields as shown further by numerical simulations. The incident fluence of the main pulse used in the present study significantly exceeds both the ablation threshold for bulk gold [39] as well as the fragmentation thresholds for gold colloidal nanoparticles in water [40–41]. It should be stressed out that the increase of the effective size of the laser spot due to filamentation can significantly reduce the incident fluence [3–4]. However, it is reasonable to assume that under our experimental conditions the fragmentation threshold will be easily reached resulting in breaking up of the gold nanoparticles into smaller clusters. Fragmentation can proceed via explosive ablation at the characteristic time scales from few tens to few hundred of picoseconds [12,42–43]. The electrostatic scenario of rupture of the particles by the repulsive Coulomb forces resulting from ejection of the hot electrons absorbing the laser irradiation was shown to dominate for particles smaller than 60 nm [12].

In this study, the laser fluence reaching the gold nanoparticles in water is much higher than 0.2 J/cm^2^, which is also close to the threshold of melt-reshaping of nanoparticles and bubble initiation [44]. Air breakdown at the close-to-film regions and filamentation of the laser pulse takes place, which obscures exact knowledge of the laser fluence at the nanoparticle location. The density of nanoparticles in the solution corresponds to the situation when a pressure build-up around the neighboring nanobubbles hinders their growth as observed from acoustic studies [45]. The 2 ns transient was observed in photo-acoustic excitation experiments using the same density of nanoparticles in solution under two-pulse irradiation, while the ebergy off the main pulse was only 100 μJ [34], comparable with the energy used for the pre-pulse in this study.

**Figure 4:**
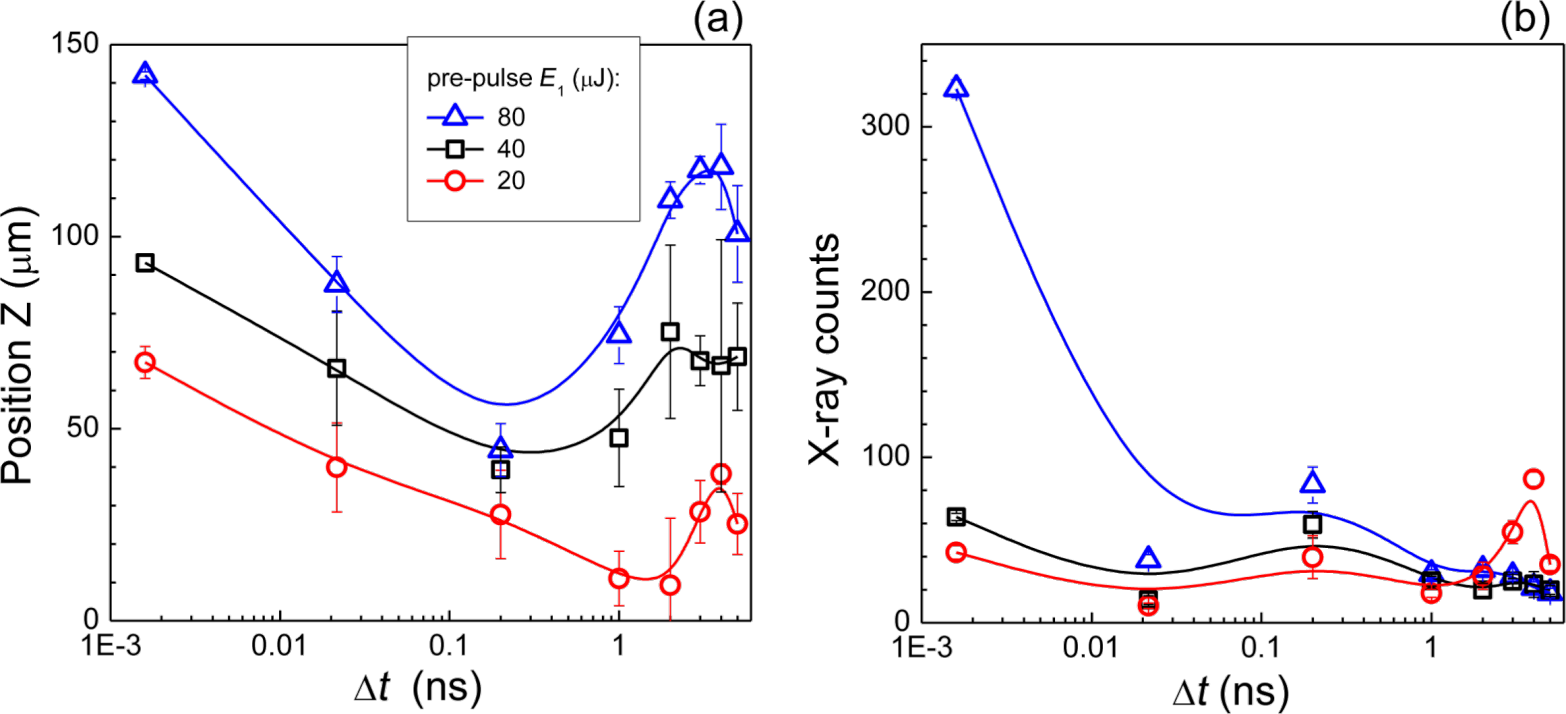
Position of X-ray maximum generation (a) and its intensity (b) for distilled water films at short excitation times and at different pre-pulse energies *E*_1_, while the main pulse energy was fixed at *E*_2_ = 700 μJ. A positive *z*-shift corresponds to an upward movement of solution position towards the incoming laser pulse (see Figure 2a). The lines are guides to the eye.

It is reasonable to assume, that the maximum intensity of X-ray emission occurs when the temporal and spatial excitation of film solutions maximizes the volume of ε_r_-near-zero (ENZ) regions and overlaps with the focal volume of the main pulse. Those ENZ regions are responsible for the most efficient energy deposition via absorption and define the transition of the propagating light to the ENZ surface (an optical near-field). This localization of light is affected by a complex pattern of surface nanoroughening of the liquid surface [28] and an internal pattern of nanobubbles. Figure 4a shows the temporal evolution of the position for maximum X-ray emission of the distilled water film consistent with a roughening of the liquid surface [28], where a positive *z*-shift corresponds to a better spatial overlap of the laser focus and the excited ENZ region on the film. A higher energy pre-pulse caused a faster evolution of maximum X-ray intensity at smaller *z*-shifts. The peak intensity of X-rays in water was on a picosecond time scale (Figure 4b) consistent with thermalization of electrons and ions [6].

### Ponderomotive action and light field enhancement

The formation of surface waves via capillary driven instabilities occurring at longer times (nanoseconds) is affected by the linear momentum transferred to the film surface, which as be calculated from the force *F* = Δ*p*/Δ*t* where the momentum in a medium of refractive index *n* of a laser pulse with energy *E* is *p* = *nE*/*c*[46]. When a light pulse of power *P* traverses from a low refractive index (*n*_0_ = 1, air) into a medium with larger refractive index (*n*_1_ = 1.33, water), momentum is gained and the force acting onto the front surface is [46]:

[4]
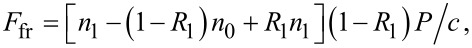


where *R*_1_ = (*n*_1_ − *n*_0_)^2^/(*n*_1_ + *n*_0_)^2^ = 0.02 is the reflection coefficient. One finds for a pulse of *E*_p_ = 100 μJ of *t*_p_ = 40 fs that *F*_fr_ = −2.32 N (along the *z*-axis), where the sign indicates that the force acting onto the surface is in the opposite direction to the laser pulse propagation (opposite to the *z*-axis in this study). The force acting on the rear surface of the solution jet can be estimated from:

[5]
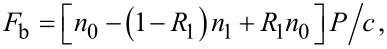


which is *F*_b_ = 3.14 N, i.e., a pushing force along the light propagation direction. Considering a breakup of the incoming high intensity laser pulse into separate microfilaments, the transfer of momentum into the solution film surface should be one of the mechanisms [3–4] resulting in the formation of surface nanoroughening and surface waves leading to droplet formation at longer times. Different scenarios are encountered by the main pulse depending on the pre-pulse excitation of the solution film. When the pre-pulse energy increases, the main pulse encounters a strongly excited ENZ shell around the gold nanoparticles as well as, at longer delays, surface roughening of the solution film and cavitation bubbles below the surface [47]. Such scenarios are simulated in the following using the finite-difference time-domain (FDTD) method (Lumerical).

**Figure 5:**
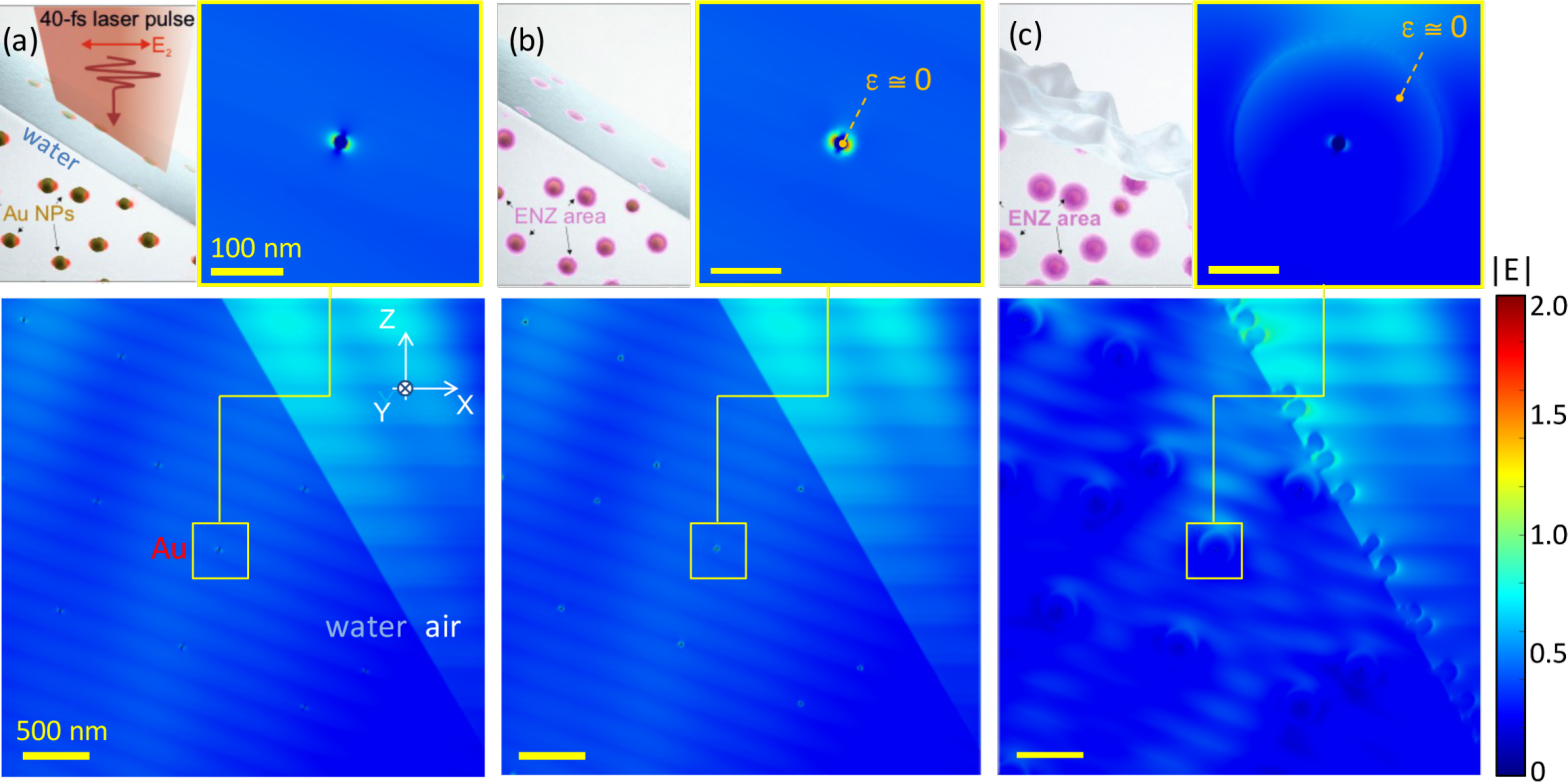
FDTD simulations of light-field enhancement under the conditions of the experiment: angle of incidence 60°, 800 nm wavelength, the concentration of gold nanoparticle yields a separation of ca. 1 μm between them. (a) Interaction of a water film with 20 nm-diameter gold nanoparticles and a laser pulse (modeled as a plane wave incidence). (b) The formation of a plasma around nanoparticles with permittivity near zero ε ≈ 0.86 + i0.86 (ENZ) in which light is efficiently absorbed: 20 nm wide shell around a 20 nm diameter nanoparticle. (c) Sphere of strongly ionized water around a gold nanoparticle. The absorption depth under ENZ plasma conditions is *l*_s_ = 149 nm, which is taken as radius of the excited volume. Nanoroughening of water surface evolves at longer times. Disintegration of gold nanoparticles is expected inside the cavitation bubble. Intensity of incident beam: 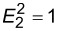.

Figure 5 shows the light-intensity distributions around gold nanoparticles modeling different stages of excitation from initial gold nanoparticles towards the formation of ENZ plasma around them. The absorption (skin) depth is defined by the imaginary part of permittivity and the ENZ conditions is *l*_s_ = λ/(2πκ) ≈ 149 nm. A large volume in which the light intensity is enhanced by up to *E*^2^ ≈ 4-times is clearly distinguished. In contrast to the case without excitation (Figure 5a), the gold nanoparticles are coated with a 20 nm wide shell of ENZ material after excitation (Figure 5b). The formation of nanoscale roughening of the liquid surface occurrs at a later stage and affects the light intensity distribution [28] in subsurface regions of the colloidal suspension film (Figure 5c). The shell of ENZ material of radius equal to the skin depth *l*_s_ shows that light is mostly reflected at the rim of this volume and is not reaching the interior of the volume (as expected). These FDTD simulations illustrate only qualitatively what the main pulse encounters in a solution film of colloidal gold nanoparticles and that a strong light-field enhancement of about four-times is expected at the gold–water interface, where the ENZ region is created.

New possibilities can be explored in applications by using the proposed hard X-ray generation under ambient conditions. Creation and localization in time and space of the high-temperature plasma as X-ray emitter is the key challenge. The most efficient energy deposition takes place in the ENZ regions. This is consistent with a high-energy plasma driven by a resonant absorption at near-critical plasma density regions [3–4,29–30]. The pump–probe concept leads to the evolution of a breakdown plasma, which is required for complex targets such as nanoparticle jets. The qualitative analysis outlined here needs detailed knowledge of the light filamentation and propagation in the regions of air breakdown close to the jet to better estimate the spatial and temporal evolution of the ENZ regions.

## Conclusion

A strong enhancement of X-ray emission from a gold nanocolloidal suspension was achieved for double-pulse excitation and could be useful for the practical implementation of X-ray generation under ambient condition. High-precision beam steering allows for a stable tracking and X-ray intensity in real time. The enhancement of X-ray emission was discussed in terms of an optimized overlap of the focal region of the laser and the solution flow, which resulted in a nanorough surface and the generation of nanobubbles leading to a strong localization (ENZ regions) and absorption of light. The fast generation (ca. 1 ns) of X-rays from the gold nanocolloidal suspension compared to distilled water (7–8 ns) was observed. The presented analysis of energy deposition into the ENZ regions is important for a detailed description of X-ray generation as well as in other fs-laser processes [48]. In this paper, spherical gold nanoparticles with a diameter of about 20 nm and a concentration of ca. 3.5 × 10^11^ nanoparticles/cm^3^ were used. Single-pulse excitation for X-ray emission [17] was carried out with spherical gold nanoparticles with different diameters from 10 to 100 nm and the results show that the appropriate size for the highest X-ray intensity is 40–50 nm. For ultrasound generation under fs-laser excitation [34,49], gold nanorod particles with more efficient surface plasmon resonance effects [50] were also used. Further enhancements of X-ray intensities are expected under double-pulse excitation with different sizes, shapes, or concentrations of nanoparticles.

It is clearly shown that the controlled spatial positioning of the solution flow at the laser focus with appropriate delay time induces effective X-ray emission. Recently, similar experiments with narrowly focussed fs-laser pulses for terahertz-wave emission from water flow were reported [51], while terahertz emission from various solutions under broad focusing was already known [52]. Simultaneous emission of X-ray and terahertz waves from gas clusters in vacuum [53–54], Al-coated glass substrates in vacuum [54], or a distilled water flow in air under double-pulse excitation [16] were independently reported. A simultaneous emission of X-ray and terahertz wave radiation is highly expected for novel pump–probe experiments [55]. The knowledge obtained in this paper will contribute to further advancements in this field.

## Acknowledgements

T.Y. thanks the partial financial support from Kakenhi (B, 18H01820) from JSPS, Japan. S.J. is grateful for partial support via the ARC Discovery DP170100131 grant, A.A.K. for the RSF no.17-19-01325 grant. The authors acknowledge discussion of light-matter interaction aspects with Prof. Eugene G. Gamaly. FDTD simulations were performed on the swinSTAR supercomputer at Swinburne University of Technology.
